# Violence detection in surveillance video using low-level features

**DOI:** 10.1371/journal.pone.0203668

**Published:** 2018-10-03

**Authors:** Peipei Zhou, Qinghai Ding, Haibo Luo, Xinglin Hou

**Affiliations:** 1 Shenyang Institute of Automation, Chinese Academy of Sciences, Shenyang, Liaoning Province, China; 2 University of Chinese Academy of Sciences, Beijing, China; 3 Key Laboratory of Opto-Electronic Information Processing, CAS, Shenyang, Liaoning Province, China; 4 The Key Lab of Image Understanding and Computer Vision, Liaoning Province, China; 5 Space Star Technology Company Limited, Beijing, China; Northeast Normal University, CHINA

## Abstract

It is very important to automatically detect violent behaviors in video surveillance scenarios, for instance, railway stations, gymnasiums and psychiatric centers. However, the previous detection methods usually extract descriptors around the spatiotemporal interesting points or extract statistic features in the motion regions, leading to limited abilities to effectively detect video-based violence activities. To address this issue, we propose a novel method to detect violence sequences. Firstly, the motion regions are segmented according to the distribution of optical flow fields. Secondly, in the motion regions, we propose to extract two kinds of low-level features to represent the appearance and dynamics for violent behaviors. The proposed low-level features are the Local Histogram of Oriented Gradient (LHOG) descriptor extracted from RGB images and the Local Histogram of Optical Flow (LHOF) descriptor extracted from optical flow images. Thirdly, the extracted features are coded using Bag of Words (BoW) model to eliminate redundant information and a specific-length vector is obtained for each video clip. At last, the video-level vectors are classified by Support Vector Machine (SVM). Experimental results on three challenging benchmark datasets demonstrate that the proposed detection approach is superior to the previous methods.

## Introduction

In public places, violent behaviors pose a serious threat to personal security and social stability. At present, millions of equipment are applied in public places, leading to a huge pressure on the security attendants. Therefore, it is of great significance to automatically detect violence events from the vast amounts of surveillance video data. For the consideration of different applications including video annotation, video retrieving and real-time monitoring, we focus on the challenging task of detecting violent activities in surveillance videos. This task involves many related computer vision techniques, for instance, object detection, action recognition and classification. Referring to the definition provided by Schedi et al. [1], we define the violent scenes as those an 8-year-old child should not watch because of physical violence. The goal of violence detection is to automatically and effectively determine whether the violence occurs or not within a short video sequence.

In the field of video-based violence detection, it is difficult to capture effective and discriminative features as a result of the variations of human body. The variations are mainly caused by scale, view point, mutual occlusion and dynamic scenes. In early attempts, most researches detected violence scenes by recognizing some violence-related characteristics like flame, blood, gunshots, explosions and car-braking [2–4]. However, this kind of method is limited by its disadvantages, such as low detection rate and high false alarm. Besides, these characteristics are not suitable for the general surveillance systems which always lack the audio information.

In recent studies about violence detection, some spatiotemporal descriptors around the interest points have received great popularity, such as STIPs [5, 6] and MoSIFT [7–9]. After that, the Bag of Words (BoW) framework [10] and a classifier such as Support Vector Machine (SVM) are adopted to distinguish the violent sequences. To recognize the human actions in surveillance videos, Chen and Hauptmann [7] designed MoSIFT descriptor, which not only encoded the local appearance but also explicitly modeled local motion. Then, a bigram model was applied to capture the co-occurrence of two video words. Considering the good performance of MoSIFT [7] and STIP [5] in action recognition, Bermejo et al. [8] applied them to assess the performance in the fight detection problem with the well-known BoW framework [6, 10, 11]. The results showed that MoSIFT and STIP performed comparably on the Hockey Fight dataset [8]. Xu et al. [9] employed MoSIFT algorithm to extract the low-level description for violent videos. To eliminate redundant features and obtain more discriminative features, the non-parametric Kernel Density Estimation (KDE) and sparse coding were exploited to select the MoSIFT descriptors and process the selected features. Then, the typical BoW model was used before classification. Senst et al. [12] proposed LaSIFT descriptor to model appearance information and Lagrangian-based motion features for violent video classification. The LaSIFT feature was evaluated with BoW framework and showed better performance than SIFT [13] and MoSIFT [7] descriptors on the Hockey Fight dataset [8] and the Crowd Violence dataset [14]. In Reference [15], a novel approach, that could effectively describe dynamic characteristics in violent videos, was reported for violence detection. By integrating the direction-based Lagrangian field measure into the SIFT descriptor, a new feature for violence analysis was developed. Then, the features were further processed by an extended BoW procedure. Similar to MoSIFT [7], Zhang et al. [16] developed a new descriptor named as MoWLD for violence detection. MoWLD combines two parts of information, a histogram of WLD describing the spatial appearance and HOF indicating the movement of interest points. Then, Zhang et al. [16] processed the descriptors in a similar means to Reference [9]. Although the descriptors extracted around the interest points could capture some appearance and motion information, they are restricted to the locations of the interest points and omit the valid information beyond the neighborhood of interest points.

There are another models for violence detection. A fast and robust framework was proposed by Zhang et al. [17] to detect and localize violent behaviors in surveillance videos. Firstly, a Gaussian Model of Optical Flow (GMOF) was proposed to extract candidate violence regions. Secondly, a novel descriptor called Orientation Histogram of Optical Flow (OHOF) was proposed in the candidate regions. At last, the descriptors were fed into a linear SVM to distinguish violent events from non-violent ones. However, the GMOF algorithm will show a low discriminative efficiency when the background is messy and dynamic. Datta et al. [18] detected violence by employing the information of motion trajectory and orientation extracted from a person limbs. The precise silhouettes is required to obtain the position of limbs, but the object segmentation is difficult due to the serious occlusion. Some other works represent violent videos by combining statistical features extracted from the spatiotemporal motion blobs, including mean, variance, standard deviation, centroid position, area, etc. [19–21]. The models with these features have the advantage of low computational complexity, but show a limited performance in classification accuracy. Deniz [22] proposed a novel method which used extreme acceleration patterns as the main feature of violent behaviors. These extreme acceleration features are efficiently estimated by applying the Radon transform to the power spectrum of consecutive frames. However, the extreme acceleration patterns are affected by the dynamic background, leading to a high false alarm.

Violence detection in crowded scenes presents more challenges due to the serious occlusion and moving crowd. Statistics of changes in the velocity flow vector magnitude for violent crowd behavior were considered in Reference [14]. These statistics, collected for short frame sequences, are represented using the Violent Flows (ViF) descriptor. ViF descriptors are then classified using linear SVM. This method provided a computationally efficient means for crowd violence detection. However, the ViF-based method performance decreased significantly in non-crowd behavior dataset. Based on ViF descriptor, a novel feature named Oriented VIolent Flows (OViF) was proposed for non-crowded violence detection in videos [23]. The OViF features describe the changes of motion magnitudes based on the statistics of motion orientations. However, this approach could not work well in crowded scenarios. Based on optical flow fields, Huang et al. [24] introduced a statistic method to detect violent crowd behaviors. This method considered the statistical characteristics of optical flow field and extracted a Statistical Characteristic of the Optical Flow (SCOF) descriptor to represent the video frames. The SCOF descriptors were then classified into either normal event or violent ones using linear SVM. However, this approach is restricted to SCOF descriptor which just models the motion information and could not capture the appearance features. In this work, we aim to develop a method that could effectively detect violent behaviors in both general scenes and crowded scenes.

With the great success of deep convolutional networks in the field of video-based action recognition such as the Temporal Segment Networks [25], some researchers developed deep neural networks for performing violent video recognition [26–30]. Dong et al. [29] proposed a novel multi-stream deep neural networks framework for person-to-person violence detection in videos. Through convolutional neural networks, three different types of violence features were extracted from raw videos, optical flow images and acceleration flow maps. Based on a Long Short Term Memory (LSTM) network, an encoding method was followed, and score-level fusion was obtained for integrating the three streams to predict the final confidence score for violence videos. Swathikiran and Oswald [30] presented an end-to-end deep neural network model to classify videos into violent and non-violent ones. This model employed the convolutional neural network to extract frame level features and then aggregated them using convolutional long short term memory (ConvLSTM). Compared with the traditional fully-connected LSTM, ConvLSTM could generate a better video representation and reduce the risk of over-fitting. Similarly, Zhou et al. [27] constructed a FightNet to represent the complicated visual violence interaction with three kinds of input modalities, i.e., RGB images for spatial networks, optical flow images and acceleration images for temporal networks. Experimental results showed the good performance in the field of violence detection. Generally, the deep neural networks for video based violence detection are pre-trained on UCF101 [31] to prevent over-fitting. However, the networks on the targeting datasets do not always perform well especially for the datasets that are greatly different from the pre-trained dataset, such as the Crowd Violence dataset [14]. In this sense, the deep learning based methods are impeded by a major obstacle: lacking a big enough training dataset for violence. Besides, it is inevitable that the deep neural networks suffer from higher computational complexity, which need more advanced hardware devices.

Summarizing the previous work and targeting the above challenges, we pay more attention to the exploration of traditional detection methods ranging from the general interactional violence to crowd violence. According to a comparative analysis on features elaborated by Lam et al. [32], experimental results demonstrated that low-level visual features and motion features played very important roles in the overall performance. In this work, we propose to extract two kinds of low-level visual features (LHOG and LHOF) from the motion regions instead of extracting descriptors around the interest points. After that, the low-level features are processed under the traditional BoW framework and then predicted by SVM classifier. Experimental results obtained on three different datasets demonstrate that the proposed method are superior to the other methods.

## Materials and methods

**Fig 1:**
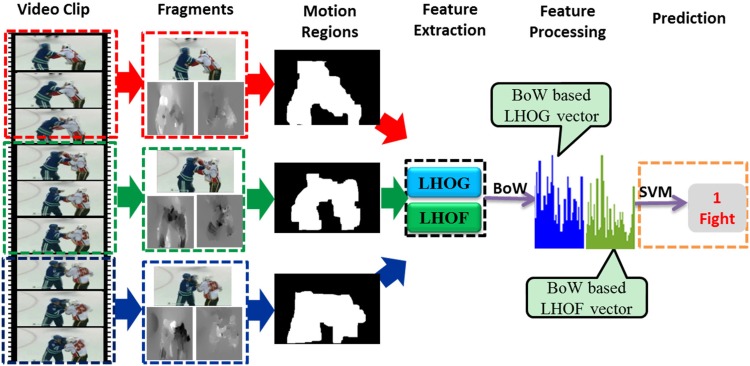
General flow chart of the proposed approach. Five phases are involved: video preprocessing, motion region segmentation, feature extraction, feature processing and prediction.

As shown in Fig 1, the general flow chart of the proposed approach is composed of five phases: video preprocessing, motion region segmentation, low-level feature extraction, feature processing and classification/prediction. As mentioned in Reference [25], consecutive frames are highly redundant, so there is no need to extract images frame by frame. During the phase of video preprocessing, we extract frames from a long video sequence using a sparse temporal sampling strategy, which is called temporal segment framework [25]. For a video clip *V*, it is equally divided into *K* segments {*S*_1_, *S*_2_, ⋯, *S*_*K*_}, and *K* short fragments {*s*_1_, *s*_2_, ⋯, *s*_*K*_} are randomly sampled from each segment. Then, the algorithm of violence detection is designed in terms of the *K* short fragments. Next, we will make details about the proposed approach.

### Motion region segmentation

For a video clip without any moving object and captured by static camera, it is easy to judge that there is no violent behavior. However, when the videos are not captured by static camera and the background is dynamic, it is difficult to extract the foreground objects using background subtraction since the moving background can hardly be modeled. By analysis, we observe that the texture of optical flow field is a strong cue of the moving objects.

**Fig 2:**
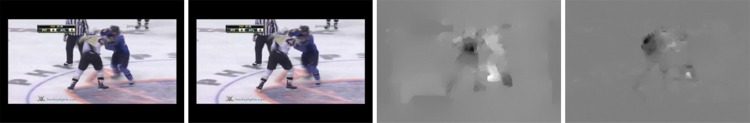
An example of optical flow images. Two consecutive frames, *flow*_*x*_ and *flow*_*y*_ of the latter frame.

Based on the distribution of optical flow fields, a motion region segmentation algorithm is proposed in this work. For the purpose of extracting optical flow fields, the TVL1 optical flow algorithm [33] implemented on OpenCV with CUDA is utilized to finish this task. As shown in Fig 2, the *x* and *y* direction optical flow images (*flow*_*x*_ and *flow*_*y*_) are computed [33] using two consecutive gray-scale images. In terms of the *flow*_*x*_ and *flow*_*y*_ images, the motion magnitude image (*Mag*) is defined in Eq 1 as follows:

$$\begin{array}{c} Mag\left(i,j\right)=\sqrt{flow_{x}\left(i,j\right)+flow_{y}\left(i,j\right)}, \end{array}$$ (1)

where (*i*, *j*) is the position of a pixel, and *flow*_*x*_ and *flow*_*y*_ denote the *x* and *y* direction optical flow images, respectively.

**Fig 3:**
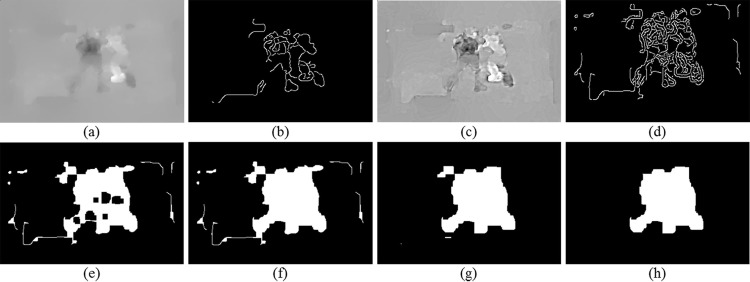
General process of motion region segmentation. (a) Motion magnitude image, (b) Canny edge image of (a), (c) sharpened motion magnitude image, (d) Canny edge image of (c), (e) closing operation on (d), (f) filling holes of (e), (g) deburring for (f), (h) segmented motion region of (a).

The edge detection algorithm using Canny operator is employed on the motion magnitude images. However, edges in optical flow images are not always obvious, as shown in Fig 3(b). To tackle with this problem, we make an enhancement on the motion magnitude image using Guided Image Filtering [34] to sharpen the image. Fig 3(c) and 3(d) show the enhanced motion magnitude image and the edge image with the same parameter as Fig 3(b). Thereon, a binary morphological image processing method, closing operation is conducted on the edge image, leading to the connectivity of motion regions, as shown in Fig 3(e). However, there is always some small holes inside the motion regions as a result of the consistent movement of some parts of the person. We simply fill the holes and consider them as parts of the corresponding motion regions, as shown in Fig 3(f). To delete the burrs of motion regions, we propose to delete the pixels in horizontal or vertical ordinate whose consecutive pixel number is lower than a threshold, 10 pixels in the experiments. Afterwards, the small region whose pixel number is lower than 150 in this paper in Fig 3(g) is viewed as noisy area and eliminated. Finally, the motion region is segmented as shown in Fig 3(h). Additionally, the images in the experiments are resized as 160 × 120.

### Low-level feature extraction

After segmenting the motion regions, we extract features from these regions. The torsos or legs in action videos are not visible in most cases due to the occlusion among people. However, the standard Histogram of Oriented Gradient (HOG) descriptor [35] was designed for human detection and could not work well in this kind of scenario. Targeting to this task, we propose to represent the actions in videos using the features of Local Histogram of Oriented Gradient (LHOG). In order to make full use of the temporal information, we put forward another descriptor (LHOF) to capture the dynamic changes. LHOG and LHOF could independently describe parts of the person and extract meaningful information from partially occluded persons, which is suitable for violence detection.

In this work, LHOG features are obtained from RGB images and LHOF features are captured from the motion magnitude images. Information from different modalities is complementary and expresses different characteristics of an action. A LHOG (or LHOF) descriptor is extracted from a “block”, which is composed of “cells”. Take the LHOG feature for example, we detail the process of feature extraction as follows.

**Input:** An image *I*, the binary motion region image *Mask*, the cell size *c*_*m*_ × *c*_*n*_, the number of orientation bins *bin*_*n*_ and the number of cells in a block *b*_*m*_ × *b*_*n*_. We denote the width and height of a block as *b*_*w*_ and *b*_*h*_, here, *b*_*h*_ = *c*_*m*_ × *b*_*m*_ and *b*_*w*_ = *c*_*n*_ × *b*_*n*_.

**Output:** LHOG features of the input image *I*.

1. **Get Valid Blocks.** The *Mask* image is scanned by a *b*_*h*_ × *b*_*w*_ pixel template with a $\frac{b_{h}}{2}$ vertical stride and a $\frac{b_{w}}{2}$ horizontal stride. The spatial region covered by the template is viewed as a block. For each block, if more than half of the pixels are located at the moving regions, the block is defined as a valid block. Record the centroid coordinates of *S* valid blocks into the matrix *Blocks*.
2. **Calculate Gradients.** Calculate the orientation and magnitude for every pixel of *I* using [−1, 0, 1] gradient filter in *x* and *y* directions, denoting as *Gradient*.
3. **Get Cell Vector.** The orientation bins are evenly spaced over 0°–360°. Every pixel votes for a 1 × *bin*_*n*_ histogram according to the orientation of *Gradient*, and the weight is the magnitude of *Gradient*. For a *c*_*m*_ × *c*_*n*_ pixel cell, the votes are accumulated into *bin*_*n*_ orientation bins. Therefore, each cell is represented as a 1 × *bin*_*n*_ row vector, *CellVector*.
4. **Get Block Vector.** For *b*_*m*_ × *b*_*n*_ cells in the *Block*, they are constructed as different *CellVector*. Combine them into a long row vector, i.e., *BlockVector* (*b*_*m*_ × *b*_*n*_ × *bin*_*n*_). Normalize the *BlockVector* according to the following formula: $BlockVector=\frac{BlockVector}{\left|BlockVector\right|}$.
5. **Get LHOG features of *I*.** A *BlockVector* is called a LHOG feature. Combine the *S* block vectors extracted from the *S* valid blocks into a matrix, named as the LHOG features of the image *I*.

**Fig 4:**
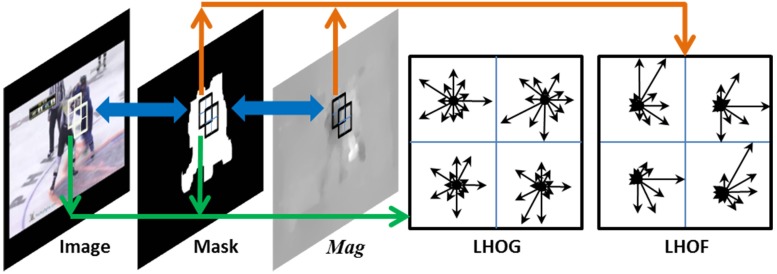
An example of low-level feature extraction, where *Mag* is the motion magnitude image. Here, a block consists of 2 × 2 cells and the block steps by half length of a block. Each cell contains 4 × 4 pixels and 12 bins are selected for each cell, forming a 48-element vector for a LHOG (LHOF) descriptor.

In this work, the feature of LHOG is extracted from an 8 × 8 pixel block. A block is composed of 2 × 2 cells and each cell contains 4 × 4 pixels. For each cell, a local histogram of oriented gradients is constructed, forming an orientation histogram with 12 dominant bins. Hence, a LHOG descriptor results in a vector of 48 (2 × 2 × 12) elements. To give a better tolerance to illumination variation and some other noises, we take two strategies: firstly, the block stride by half of itself, i.e., the overlap is half of a block; secondly, the normalization is conducted for each LHOG. Different from LHOG descriptor, LHOF is extracted from the motion magnitude images, which captures the dynamic information. Fig 4 presents an example of feature extraction.

### Feature processing

#### Bag of Words (BoW) model

BoW model has become a popular method for image classification and action recognition [6, 8, 11]. In this work, low-level features are extracted from the motion regions. However, the number and size of motion regions are different for different video clips, leading to different-length features. Under the framework of BoW model, the extracted low-level features are represented as a fixed-length vector using a histogram which reflects the frequency of different words. The visual words in the BoW model are typically defined as the cluster centers which are obtained using *k*-means clustering method over the low-level features (LHOG or LHOF). The number of visual words could be set according to the practical application requirement. Intuitively, BoW approach collects the statistic information of the feature distributions. Thereon, the vectors with the same length could be further processed using a standard classifier.

Some previous methods process features with BoW framework after fusing the features. For instance, MoWLD [16] is a long vector by directly combining HOG and HOF, followed by the BoW method. However, HOG and HOF features may not share the same class space, which will reduce the discriminative ability. Different from the previous early-fusion strategy, we make late-fusion for the extracted features. In this work, we argue that the class space of LHOG features is different from that of LHOF features. According to this argument, LHOG and LHOF features are respectively processed using the BoW model, resulting in two kinds of vectors with the same length. Then, the two kinds of vectors are combined before feeding into the classifier. Experimental results demonstrate that the late-fusion method outperforms the early-fusion method for the low-level features in this work.

In the phase of classification, we take the widely used SVM with a Radial Basis Function (RBF) kernel as the classifier to distinguish the violent video sequences. An integrated software for support vector classification, LIBSVM [36] is adopted in the experiment stage.

## Results and discussion

### Dataset

**Fig 5:**
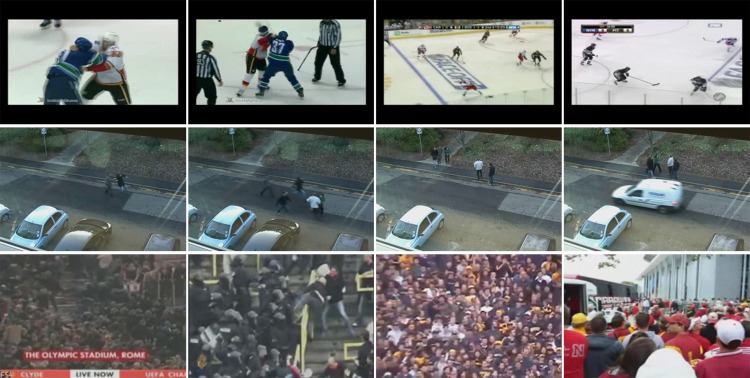
Frame examples of three datasets employed in the experiment. They are extracted from the Hockey Fight dataset (first row), the BEHAVE dataset (second row) and the Crowd Violence dataset (third row). And the left two columns list violence frames and the right two columns show non-violence samples.

In this work, experiments are carried out on three challenging datasets: the Hockey Fight dataset [8], the BEHAVE dataset [37] and the Crowd Violence dataset [14], as shown in Fig 5. The selected datasets are very representative, including videos recorded by both static and moving cameras, videos presenting the violence of a few persons and crowd violence, and videos with some other challenges such as varying scales and uneven illumination.

#### Hockey Fight dataset

This dataset is composed of 1000 video clips collected from hockey games of the National Hockey League (NHL) and all the videos are recorded by moving camera. Half of them (500 clips) are labeled as fight and another half as non-fight. Each clip roughly contains 40 frames with resolution of 360 × 288.

#### BEHAVE dataset

Shot by a static camera, five videos (640 × 480 pixels image resolution) with more than 5000 frames construct this dataset, involving group discussing, walking, running, chasing and fighting, with the disturbance of cycling and passing cars. Similar as Reference [16], we pick up 80 clips from the videos, including 20 fight samples and 60 non-fight ones. Since only 392 frames are labeled “Abnormal Interaction” in this dataset, each fight clip contains 19 frames. 80 non-fight clips are randomly selected from the frames labeled as “Normal Interaction”, 19 frames included in each clip too.

#### Crowd Violence dataset

This dataset mainly presents the crowd violence behavior and most of the scenes are dynamic, which greatly increases the detection difficulty. 246 video clips (320 × 240 pixels image resolution) are assembled on this dataset with 123 violent samples and 123 non-violent ones.

At the stage of experiment, the images are resized into 160 × 120 before extracting features.

### Experimental results

At present, the state-of-the-art techniques about violence detection involve several approaches: the BoW method, the Violent flows (ViF) method [14], the Robust Violence Detection (RVD) method [17], the MoSIFT method [8], the MoWLD method [16] and the Appearance and Motion DeepNet (AMDN) method [28]. We make comparison with the above five methods on three benchmark datasets with 5-fold cross validation test method. In this work, we adopt the following common used evaluation indexes: mean accuracy (ACC), accuracy standard deviation (SD) and the area under the ROC curve (AUC).

At the stage of video segmentation, we adopt the same parameter *K* = 3 the same as Reference [25] and 5 frames are randomly chosen in each segment, which extremely reduces the temporal redundancy. In the phase of dictionary learning, it is hard to construct the dictionary due to the huge amount of extracted features. Here, we randomly select 3% features, and then cluster them into predefined-number classes. On the BEHAVE and Crowd Violence datasets, the number of dictionary words of BoW model is set to 300.

#### Results on the Hockey Fight dataset

Table 1 summarizes the accuracy results of various methods based on the BoW model paired with HOG, HOF, MoSIFT [8], MoWLD [16] and LHOG, respectively. As shown in Table 1, based on BoW model, MoWLD descriptor obtains similar accuracy to HOG feature, and achieves a little enhancement compared with MoSIFT feature. It is obvious that the proposed LHOG descriptor performs much better than the traditional HOG [8] feature and the MoWLD [16]. Based on the BoW model, the LHOG descriptor achieves higher accuracy rate than the MoWLD descriptor [16], which indicates that the proposed LHOG and LHOF are of great efficiency and discrimination. Intuitively, LHOG features only represent the appearance information of the video sequence. However, LHOG achieves better performance than some spatio-temporal descriptors such as MoSIFT and MoWLD within the framework of BoW model as shown in Table 1. Therefore, it can be concluded that LHOG descriptor is a very discriminative feature for violence detection. Another interesting conclusion could be drawn from the last column of Table 1 that the accuracy rate does not always increase with the increase of dictionary size. Therefore, it is of vital significance to select an appropriate an appropriate dictionary size to balance the good performance and low computation complexity.

**Table 1:** Comparison of accuracy rate based on BoW method on the Hockey Fight dataset.

Vocabulary	Existing				Proposed
	HOG [8]	HOF [8]	MoSIFT [8]	MoWLD [16]	LHOG
**50**	87.80%	83.50%	87.50%	88.10%	**93.40%**
**100**	89.10%	84.30%	89.40%	90.40%	**94.30%**
**150**	89.70%	85.90%	89.50%	90.70%	**94.50%**
**200**	89.40%	87.50%	90.40%	91.30%	**94.90%**
**300**	90.80%	87.20%	90.40%	91.30%	**95.00%**
**500**	91.40%	87.40%	90.50%	91.50%	**94.80%**
**1000**	91.70%	88.60%	90.90%	91.90%	**94.60%**

The first column is the dictionary size of BoW model, i.e., the word number of the dictionary. Using the same feature processing method (BoW), LHOG descriptor achieves the best performance on the challenging Hockey Fight dataset.

In addition to LHOG descriptor, we propose to extract LHOF descriptor to capture the dynamic information. LHOG and LHOF features are complementary spatio-temporally for the representation of violent behaviors. The detailed comparison results are listed in Table 2 between the proposed features (LHOG+LHOF) and the MoWLD descriptors [16]. Due to the large dimension of MoWLD (1536), Zhang et al. [16] employed the KDE-based feature selection and sparse coding approach to remove the irrelevant and redundant features. Then the BoW model was applied into the refined features. For “MoWLD+KDE+ SparseCoding” method [16] in Table 2, the number of vocabulary words of BoW model is set to be the same as the dictionary size of sparse coding. With respect to the indices of ACC and SD on the Hockey Fight dataset, the proposed low-level features perform generally better than the MoWLD descriptor while they are evenly matched with the AUC index (0.9798 vs. 0.9789).

**Table 2:** Accuracy comparison of MoWLD using KED and sparse coding method and proposed features based BoW model on the Hockey Fight dataset.

Vocabulary	Existing		Proposed	
	MoWLD+KDE+SparseCoding [16]		LHOG+LHOF	
	ACC±SD	AUC	ACC±SD	AUC
**50**	91.4±1.78%	0.9597	93.6±1.14%	0.9725
**100**	92.9±2.18%	0.9615	94.4±1.38%	0.9756
**150**	93.9±1.84%	0.9695	94.7±1.87%	0.9800
**200**	94.7±1.62%	0.9715	94.9±2.10%	0.9823
**300**	94.6±1.71%	0.9708	**95.1±1.15%**	**0.9798**
**500**	**94.9±1.68%**	**0.9789**	94.8±1.48%	0.9800
**1000**	94.2±1.91%	0.9719	94.7±1.67%	0.9805

“LHOG+LHOF” denotes the combination of LHOG and LHOF descriptor after feature processing. The vector length of a “LHOG+LHOF” descriptor is double of the dictionary size, for instance, 1 × 600 when “Vocabulary” is 300. The proposed approach makes a slight progress compared with the “MoWLD+KDE+SparseCoding” method.

#### Results on the BEHAVE dataset

It is a relatively simple dataset recorded with a static camera and the challenges of violence detection mainly come from the similar actions such as running. For the sake of demonstrating the superiority of the proposed method, it was compared with the state-of-the-art approaches, including MoWLD [16], MoSIFT [8], HNF (combination of HOG and HOF) [14], ViF [14], RVD [17] and AMDN [28]. Besides the proposed low-level features, Table 3 lists the results of various violence detection models on the BEHAVE dataset. As shown in Table 3, compared with the previous methods, “LHOG+LHOF+BoW” method achieves the best result with the accuracy up to 100%. The reasons of the good performance are summarized as follows. Firstly, the dataset is recorded by a static camera and the scene is relatively simple. Secondly, the proposed method of motion region segmentation has filtered out most of the background interference. Thirdly, the fight clips are distinctly different from the non-fight clips. Last but not the least, the LHOG and LHOF features are more effective to detect violence behaviors compared with the previous algorithms. The large SD of “LHOF+BoW” method mainly results from the low discriminative efficiency between the violence and the fast running in optical flow images.

**Table 3:** Results of violence detection on the BEHAVE dataset.

Algorithm		ACC±SD	AUC
**Existing**	**HOG+BoW** [14]	58.69±0.35%	0.6322
	**HOF+BoW** [14]	59.91±0.28%	0.5893
	**HNF+BoW** [14]	57.97±0.31%	0.6089
	**ViF** [14]	82.02±0.19%	0.8592
	**MoSIFT+BoW** [8]	62.02±0.23%	0.6578
	**RVD** [17]	85.29±0.16%	0.8878
	**AMDN** [28]	84.22±0.17%	0.8562
	**MoWLD+BoW** [16]	83.19±0.18%	0.8517
	**MoWLD+SparseCoding** [16]	85.75±0.15%	0.8891
	**MoWLD+KDE+SparseCoding** [16]	87.17±0.13%	0.8993
**Proposed**	**LHOG+BoW**	**100±0.00%**	**1.0000**
	**LHOF+BoW**	97.50±3.42%	0.9875
	**LHOG+LHOF+BoW**	**100±0.00%**	**1.0000**

“LHOG+LHOF+BoW” denotes the detection method using “LHOG+LHOF” descriptor combined with BoW model.

#### Results on the Crowd Violence dataset

It is the most challenging dataset among the selected three datasets owing to the messy crowd. In Table 4, several state-of-the-art violence detection algorithms are implemented to demonstrate the efficiency of the proposed approach. When comparing the performance on the BEHAVE dataset with that on the Crowd Violence dataset, we find that the accuracy rates of MoSIFT [8] and RVD [17] methods decrease slightly, while the ViF [14] and AMDN [28] approaches remain relatively stable on different datasets. As it can be seen from Table 4, compared with the previous methods, the proposed “LHOG+LHOF+BoW” method achieves much higher accuracy but with a slight higher standard deviation (SD). By analysis, most of the false alarms result from people’s fast running or quick moving camera, and some missed alarms are caused by the disturbance of the chaotic crowd. Besides, Table 4 presents that the “LHOG+LHOF+BoW” improves a lot on the “LHOG+BoW” method, demonstrating the importance of LHOF features for crowd violence detection. Experimental results on this dataset illustrate that the proposed approach could detect violent behaviors in crowded scenes with high efficiency.

**Table 4:** Results of violence detection on the Crowd Violence dataset.

Algorithm		ACC±SD	AUC
**Existing**	**HOG+BoW** [14]	57.43±0.37%	0.6182
	**HOF+BoW** [14]	58.53±0.32%	0.576
	**HNF+BoW** [14]	56.52±0.33%	0.5994
	**ViF** [14]	81.30±0.21%	0.8500
	**MoSIFT+BoW** [8]	57.09±0.37%	0.6073
	**RVD** [17]	82.79±0.19%	0.8659
	**AMDN** [28]	84.72±0.17%	0.8891
	**MoWLD+BoW** [16]	82.56±0.19%	0.8651
	**MoWLD+SparseCoding** [16]	86.39±0.15%	0.9018
	**MoWLD+KDE+SparseCoding** [16]	89.78±0.13%	0.9472
**Proposed**	**LHOG+BoW**	89.84±1.76%	0.9461
	**LHOF+BoW**	86.57±1.91%	0.9039
	**LHOG+LHOF+BoW**	**94.31±1.65%**	**0.9703**

The performance of “LHOG+LHOF+BoW” improves a lot on the “LHOG+BoW” method, demonstrating the importance of LHOF features for crowd violence detection.

### Discussion

In this work, the important parameters mainly involves the phases of video segmentation, feature extraction and dictionary construction in the BoW model. We divide a video into *K* (*K* = 3) segments, and *s* (*s* = 5) frames are randomly selected in each segment. Experimental results show that there is little difference when we increase *K* or *s*. In the phase of feature extraction, a block contains 2 × 2 cells, a cell is set to 4 × 4 pixels, and an orientation histogram with 12 bins is formed for each cell. We found that these parameters yielded the best performance after we considered other block size (e.g. 4 × 4 cells) and cell size (e.g. 4 × 8 and 8 × 8). The number of histogram bins plays an important role in the detection system and it is set to 12 to balance the accuracy and computational complexity. In the phase of feature processing by BoW model, dictionary construction is the most time-consuming step, which is positively related with the dictionary size. However, the performance does not keep improving with the dictionary size increasing. Therefore, an optimal value could be obtained according to the detection accuracy.

Comparison with the traditional HOG features. Targeting to the task of human detection, the original HOG feature is a global feature extracted from the whole image and could express the spatial position relationships between the body parts. It performs well for human detection when the person roughly keeps upright, allowing some subtle body movements. However, the target of violence detection is different from that of human detection. Firstly, the global features (HOG) inevitably introduce the irrelevant background noises. Based on this, we propose a new method to extract the motion regions in order to reduce the influence from the background noises. Secondly, when the violence behaviors happen, the actions of the objects are complex and changeable. It may not fully express the violence sequence if only one global HOG feature is extracted for each image. Based on this, we extract the local features, LHOG to express the violence behaviors. Complementarily, LHOF features are extracted to capture the temporal information. As local low-level features, LHOG and LHOF could be more flexible to express the local deformation of a target and easier to distinguish the violence features from the disturbance features. Thirdly, the BoW model is used to deal with the LHOG and LHOF features, representing the extracted features in the form of statistical information. It neglects the spatial location and temporal sequence of the low-level features, which weakens the fixed form of violence and is more suitable to express the diversity of violence. In summary, the newly proposed LHOG + LHOF features play an important role in the violence detection as well as the other processing phases.

When comparing the proposed low-level features with the quite advanced descriptor MoWLD [16], we could find the differences as follows. Firstly, the former (proposed low-level features) is extracted from the motion regions while the latter (MoWLD descriptor) is obtained around the interesting points. Intuitively, the proposed features could capture more appearance and dynamic information. Secondly, the dimension of the former (48) is far less than that of the latter (1536). Too many elements introduce a lot of irrelevant and redundant information, leading to the weak ability of violence detection. Thirdly, the former directly adopts the BoW model while the letter applies the KDE-based feature selection and sparse coding approach to remove the irrelevant and redundant features before employing the BoW model. In this sense, the computation complexity of the proposed approach is lower.

In this work, we also considered other low-level features, such as texture and statistical features of the motion regions [21]. However, the accuracy rate of the detection system with these features is lower than that without these features. We speculate that the LHOG and LHOF features could capture the texture and statistical information of the frames, so the newly added features introduce both the valid features and invalid noises, which results in a worse performance.

In brief, two kinds of low-level features, LHOG and LHOF are complementary spatio-temporally, constructing the advanced descriptors of violence detection system.

## Conclusion

The contributions of this work are summarized as follows:

1. In face of the noisy moving scenes, a new method is proposed to segment the motion regions according to the distribution of optical flow fields. The segmentation of the motion regions plays an important role in simplifying the features and decreasing the noises.
2. In the motion regions, we propose to extract two kinds of low-level features: Local Histogram of Oriented Gradient (LHOG) and Local Histogram of Optical Flow (LHOF) to represent the video-based activities spatio-temporally. LHOG could capture the appearance information and LHOF obtains the dynamic information of the objects.
3. Considering the different class spaces for different kinds of low-level features, we adopt the late-fusion strategy. That is to say, LHOG and LHOF features are processed respectively under the framework of BoW model, and then the two kinds of vectors are combined into new vectors, followed by the SVM classifier.

Compared with the previous methods, the proposed method achieves better performance on the three challenging datasets. Experimental results could practically demonstrate the effectiveness of the proposed approach for both general violence and crowd violence sequences.
